# Leukotriene Receptors: Crucial Components in Vascular Inflammation

**DOI:** 10.1100/tsw.2007.187

**Published:** 2007-09-01

**Authors:** Magnus Bäck

**Affiliations:** Department of Cardiology Center for Molecular Medicine, Karolinska University Hospital, L8:03, 171 76 Stockholm, Sweden

**Keywords:** atherosclerosis, eicosanoids, endothelium, intimal hyperplasia, leukotriene, receptor antagonists, lipoxygenase, vascular smooth muscle cells

## Abstract

The accumulation of immune cells during vascular inflammation leads to formation of leukotrienes (LTs). While macrophages represent a major source of LT biosynthesis in the proximity of the vascular wall, activated T lymphocytes may, in addition, play a key regulatory role on macrophage expression of LT-forming enzymes. Within the vascular wall, LTs activate cell surface receptors of the BLT and CysLT subtypes expressed on vascular smooth muscle and endothelial cells. The LT receptor expression on those cells is highly dependent on transcriptional regulation by pro- and anti-inflammatory mediators. LT receptor activation on vascular smooth muscle cells is associated with both directly and indirectly induced vasoconstriction, as well as intimal hyperplasia through stimulation of migration and proliferation. On the other hand, endothelial LT receptors induce vasorelaxation and leukocyte recruitment and adhesion. Results from *in vitro* and* in vivo* studies of LT receptor antagonists indicate potential beneficial effects in atherosclerosis and other inflammatory cardiovascular diseases.

### INTRODUCTION

#### Leukotriene Receptors

Structural differences divide the leukotrienes (LTs) into two separate classes: first, LT $C_4$, $D_4$, and $E_4$, referred to as the cysteinyl-LTs (cysLTs) based on the cysteine residue in the structure; second, $LTB_4$, which is structurally different, containing two hydroxyl groups instead of amino acids[1]. The cysLTs and $LTB_4$ display distinct functional characteristics. Initially, $LTB_4$ was described as a mediator of neutrophil chemotaxis, whereas the cysLTs mainly were associated with bronchoconstriction and asthma. However, all LTs induce profound effects also within the vascular wall, and the present review will focus on the consequences of LT receptor activation in vascular smooth muscle cells (SMCs) and endothelial cells (ECs).

LTs exert their actions through seven-transmembrane G-protein coupled receptors consisting of two subclasses: BLT receptors, activated by $LTB_4$, and CysLT receptors, activated by the cysLTs[2]. The BLT receptors are denoted $BLT_1$ and $BLT_2$, based on their affinity for the agonist[3]. On the other hand, the receptors activated by the cysLTs are characterized based on their sensitivity to antagonists developed for inhibition of LT-induced bronchoconstriction and are referred to as $CysLT_1$ and $CysLT_2$[4].

### LT Formation within the Vascular Wall

The first step in LT biosynthesis from arachidonic acid (AA) by the action of the enzyme 5-lipoxygenase (5-LO) and its activating protein (FLAP) leads to the formation of $LTA_4$. This reaction has, in general, been considered restricted to myeloid cells, such as granulocytes, macrophages, and mast cells[5]. However, 5-LO expression has also been detected in ECs, suggesting endothelium-dependent LT formation from AA also in the absence of leukocytes[6,7]. Moreover, the next step in cysLT synthesis, i.e., conjugation of $LTA_4$ with glutathione, can also take place in structural cells of the vascular wall as well as in platelets. Taken together, both vascular SMCs and ECs can be implicated in LT biosynthesis, potentially leading to para- and autocrine LT receptor signaling within the vascular wall.

Although macrophages represent a major source of 5-LO in the cardiovascular system[8,9], LT synthesis may also involve cells of adaptive immunity[10]. A mouse model combining hyperinflammation and -lipidemia, through selective abrogation of T-cell transforming growth factor (TGF)β signaling in $ApoE^{-/-}$ mice[11], has uncovered a stimulatory effect of activated T cells on LT synthesis mediated through up-regulation of FLAP expression in macrophages[10]. Since $LTB_4$ is a potent chemoattractant for T cells[12], the increased $LTB_4$ formation, which is detected after *ex vivo* stimulation of the aorta from these mice, will induce a vicious circle involving further T-cell recruitment and a maintained stimulation of LT synthesis in the proximity of the vascular wall, as illustrated in Fig 1. The significance of this proinflammatory T-cell/macrophage cross-talk in vascular inflammation was demonstrated by the decreased atherosclerosis in $ApoE^{-/-}$ mice lacking TGFβ signaling in T cells after treatment with an inhibitor of FLAP[10].

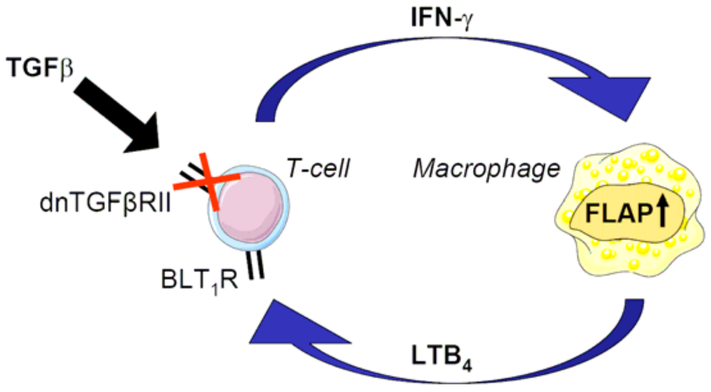

**FIGURE 1.** Schematic drawing of the macrophage/T-cell cross-talk inducing increased $LTB_4$ synthesis through up-regulation of FLAP expression[10]. The dampening effect of TGFβ on T cells was disrupted by a dominant-negative (dn) TGFβRII under the control of the CD4 promoter, resulting in increased T-cell activation and interferon (IFN)-γ production. The elevated $LTB_4$ concentrations secreted from the vascular wall induce chemotaxis and recruitment of further T cells through activation of the T-cell $BLT_1$ receptor[10].

LT formation can be detected in supernatants of *ex vivo* incubated human atherosclerotic lesions derived from both carotid[13] and coronary arteries[14]. In addition, increased cysLT concentrations are detected in urine samples from patients with unstable angina and myocardial infarction[15], as well as before and after coronary artery bypass surgery[16]. Finally, it was recently discovered that subjects with high levels of cysLTs in gingival crevicular fluid have an increased carotid artery wall thickness, regardless of the dental status[17]. While the latter reports provide circumstantial evidence for a role of LTs in cardiovascular disease, mechanistic studies have also shown that LT receptor activation can induce pathophysiological alterations within the vascular wall[18].

### BLT RECEPTORS

#### BLT Receptor Subtypes

The $LTB_4$-induced neutrophil chemotaxis takes place at the nanomolar concentration ranges, whereas the release of lysosomal enzymes is stimulated by $LTB_4$ at micromolar concentrations[19]. In addition, binding studies have revealed both high- and low-affinity binding sites for $LTB_4$ in human neutrophils[20]. Taken together, these observations have led to the hypothesis of two distinct BLT receptors, and subsequent studies cloned cDNA encoding a high-affinity $BLT_1$ receptor[21] as well as a low-affinity $BLT_2$ receptor[22,23,24,25].

Membrane fractions of Cos-7 cells transfected with the $BLT_1$ receptor show specific $LTB_4$ binding with a $K_d$ of 0.15 n*M*[21], whereas binding to the recombinant human $BLT_2$ receptor has been reported with approximately 100-fold higher $K_d$ values[22]. The notion of the $BLT_2$ receptor as a low-affinity $LTB_4$ receptor has also been supported by functional studies. In CHO cells transfected with the human $BLT_1$ receptor, the dose dependency for $LTB_4$ chemotaxis is bell shaped with an optimum concentration of 1–10 n*M*, whereas the corresponding concentration of $LTB_4$ for $BLT_2$-mediated chemotaxis is higher[21,24]. In addition, CHO cells coexpressing both BLT receptors migrate towards both low and high concentrations of $LTB_4$ (1 n*M* – 10 $\mu M$), while CHO cells expressing a single receptor only responded to a narrow range of $LTB_4$ concentrations[26].

In addition to the cell surface $BLT_1$ and $BLT_2$ receptors, $LTB_4$ may also be an endogenous activating ligand for the nuclear peroxisome proliferator activated receptor $\alpha$ (PPAR$\alpha$)[27]. However, the role of $LTB_4$-induced PPAR$\alpha$ activation in the vascular wall is presently unknown.

#### Transcriptional Regulation of BLT Receptor Expression

In vascular SMCs, $BLT_1$ receptor expression is up-regulated by lipopolysaccharide (LPS), interleukin-1$\beta$ (IL-1$\beta$), and interferon-$\gamma$ (IFN-$\gamma$), as demonstrated by studies of cells derived from human, rat, or mouse vessels[28,29]. The LPS- and IL-1$\beta$–induced increase in $BLT_1$ receptor mRNA levels in SMCs is prevented by adenoviral infection with a dominant-negative form of I$\kappa$B kinase $\beta$[28]. The latter procedure abrogates nuclear factor $\kappa$B (NF-$\kappa$B) signaling, a pathway associated with proinflammatory alterations of the vascular wall in atherosclerosis[30]. Furthermore, vascular injury has been shown to activate NF-$\kappa$B signaling[31], and SMCs isolated from the intimal thickening of the rat carotid artery after angioplasty express significantly higher levels of $BLT_1$ receptor mRNA compared with cells derived from uninjured arteries[28]. Furthermore, intimal cells transfected with the dnIKK$\beta$ construct do not exhibit increased $BLT_1$ receptor mRNA levels, suggesting the NF-$\kappa$B pathway as the main inducer of $BLT_1$ receptor transcription in the intima[28].

In line with the finding in vascular SMCs, the human $BLT_1$ promoter has been shown to contain consensus sequence for NF-$\kappa$B binding[32]. However, the transcriptional regulation of the $BLT_1$ receptor gene may vary between cells of hematopoietic and nonhematopoietic origin. For example, $BLT_1$ receptor expression is down-regulated by proinflammatory stimuli in human leukocytes, whereas anti-inflammatory mediators, such as *all-trans* retinoic acid[21], dexamethasone, and IL-10, up-regulate $BLT_1$ receptor expression in these cells[33,34]. Furthermore, in monocytic THP-1 cells, deletion of a Sp1 binding site in the $BLT_1$ receptor promoter markedly decreases transcriptional activity[32]. While Sp1

binding to the $BLT_1$ promoter may be required for basal transcription, it can most probably not explain the tissue- and cell-specific expression of the $BLT_1$ receptor[32]. The latter phenomenon may rather be dependent on methylation of the promoter region, which has been shown to regulate $BLT_1$ receptor transcription *in vitro*[32]. In this context, it is also of interest to note that another open reading frame (ORF) has been found to overlap the promoter region of the $BLT_1$ receptor gene[32]. The homology to the $BLT_1$ receptor gene identified this ORF to encode the second receptor for $LTB_4$, $BLT_2$[22].

Taken together, the above studies support a cell-specific BLT receptor transcription, in which inflammatory stimuli may result in $BLT_1$ receptor down-regulation in inflammatory cells and up-regulation in the vascular wall. It is possible that in an early phase of inflammation, the predominant effects of LTs are exerted on leukocytes. As inflammatory circuits increase, $BLT_1$ receptor down-regulation in leukocytes could serve to limit the $LTB_4$-induced effects on leukocytes. In contrast, at those later stages in the inflammatory process, $BLT_1$ receptors are up-regulated in SMCs, potentially enhancing $LTB_4$-induced responses in the vascular wall. This notion of distinct LT-induced effects on leukocytes and the vascular wall is supported by a study of sepsis induced by cecal ligation in mice. Although either 5-LO knock out or FLAP inhibition reduced neutrophil accumulation and increased the number of bacteria in the peritoneal cavity, those mice were protected against lethal sepsis-induced hypotension[35]. Even though the latter study mainly addressed cysLT formation, the results support that structural vascular cells may be the preferential targets for LT-induced effects in cardiovascular disease. Since increased $BLT_1$ receptor mRNA levels have been associated with enhanced $LTB_4$-induced vascular responses[36], a proinflammatory up-regulation of BLT receptors may hence be one possible mechanism of enhancing vascular inflammation.

### CysLT RECEPTORS

#### CysLT Receptor Subtypes

Although the nomenclature of the CysLT receptors were based solely on functional findings[4], the subsequent cloning and expression of the recombinant human CysLT receptors support the classification of the subtypes as $CysLT_1$ and $CysLT_2$[37,38,39,40,41]. In brief, early studies in guinea pig airways identified $LTD_4$-induced responses as being sensitive to the available LT receptor antagonists, whereas the latter drugs did not alter the $LTC_4$-induced contractions[4]. Since the $CysLT_1$ receptor antagonists potently inhibited the contractions to all cysLTs in human airways, the main focus of these drugs has hitherto been in the treatment of asthma[42,43]. In contrast to the airways, contractions of isolated human vascular preparations were initially shown to be resistant to the $CysLT_1$ receptor antagonists developed for treatment of asthma, but inhibited by the dual $CysLT_1$/$CysLT_2$ receptor antagonist BAY u9773[44]. A hypothesis of distinct airway and vascular CysLT receptor is, however, not generally applicable since there are also vascular segments in which cysLT-induced vasoconstriction is inhibited by the antiasthmatic drugs[45,46,47], which will be discussed in greater detail below. Finally, the limited effects of both $CysLT_1$ and $CysLT_2$ receptor antagonism in some vascular preparations have raised arguments for further CysLT receptor subtypes in the vasculature[4,48,49,50,51,52,53].

The rank order of potency for calcium mobilization in cells transfected with the human $CysLT_1$ receptor is $LTD_4$ > $LTC_4$ > $LTE_4$[37,38,40,41], with $LTE_4$ being a partial agonist[38,40]. In cells expressing human recombinant $CysLT_2$ receptor protein, the rank order of agonist potency is $LTC_4$ = $LTD_4$ > $LTE_4$, with $LTE_4$ again being partial agonist[39,40,41]. The latter findings are similar to functional results with human pulmonary venous smooth muscle, which contains a homogenous $CysLT_2$ receptor population[44]. Furthermore, the unselective CysLT receptor antagonist BAY u9773 has been identified as a selective $CysLT_2$ receptor agonist with the same potency and efficacy as $LTE_4$, but without any agonistic activity at the human $CysLT_1$ receptor[41]. Finally, BAY u9773 has been reported to induce contractions of the human pulmonary vein[44,49], indicating a usefulness of this pharmacological tool in lack of selective $CysLT_2$ receptor antagonists.

### Transcriptional Regulation of CysLT Receptor Expression

The human gene encoding the $CysLT_1$ receptor is located on chromosome Xq13-q21[37,38]. The putative promoter sequence has revealed STAT6 consensus elements, responsive to IL-4[54]. $CysLT_1$ receptor transcription is time and dose dependently increased in THP-1 cells after IL-4 stimulation, with maximum expression levels at 6 and 24 h for mRNA and surface protein expression, respectively[54]. In line with the $BLT_1$ receptor, $CysLT_1$ receptor transcription may also be NF-κB dependent, as suggested by the increased $CysLT_1$ receptor mRNA levels detected in human umbilical vein endothelial cells (HUVECs) after prolonged (>6 h) incubation with IL-1β[55]. The human $CysLT_2$ gene has been mapped to chromosome 13q14.2-21.1[39,40], but the promoter activity remains to be established. However, IL-4 also seems to play a key role in the regulation of $CysLT_2$ receptor transcription in HUVECs[56].

## LT RECEPTORS ON VASCULAR SMC

### LT Receptor Expression and Signaling in Vascular SMCs

Immunohistochemical stainings of human arteries have revealed expression of BLT1 receptor proteins in the muscular layers of carotid atherosclerotic endarterectomies, as well as in human nonatheroclerotic mammary arteries (Fig. 2)[28]. However, In human coronary artery SMCs, BLT1 receptor mRNA levels are eightfold higher compared with BLT2 receptor mRNA determined by RT-PCR[29]. The expression of BLT1 receptor proteins on human coronary artery SMCs has also been demonstrated by Western blot[28] and flow cytometry[29]. In contrast to this apparent constitutive BLT1 receptor expression in human coronary artery SMCs, murine aortic SMCs express BLT1 receptor mRNA only after 24-h treatment with tumor necrosis factor (TNF)- and IFN- [29]. The signaling pathways activated by LTB4 in the murine SMCs after BLT1 receptor induction involve increased focal adhesion kinase (FAK) activation and suppressed extracellular signal-regulated kinase (ERK) phosphorylation, as demonstrated by Western blot after 5–60 min stimulation[29]. The latter signaling pathways are involved in migration and proliferation, respectively (see below).

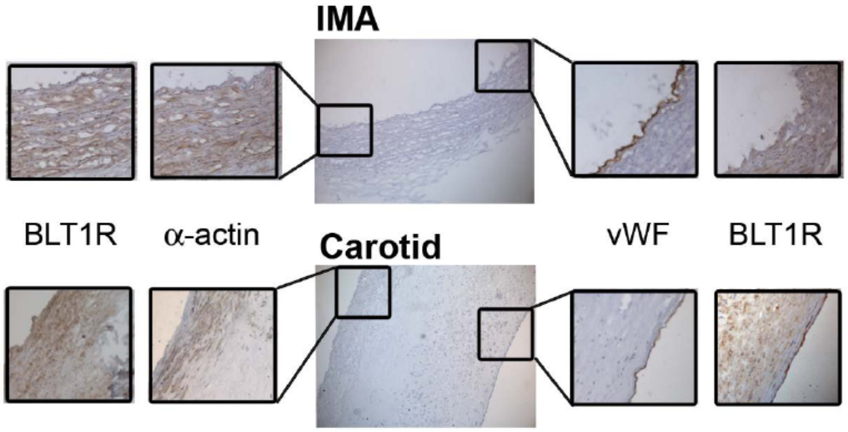

**FIGURE 2.** Immunoperoxidase staining for the $BLT_1$ receptor (BLT1R) in human internal mammary artery (IMA, top panels) and atherosclerotic carotid endarterectomy (bottom panels). $BLT_1$ receptor expression colocalized with α smooth muscle actin in both vessels, and with endothelial von Willebrand factor (vWf) exclusively in atherosclerotic lesions. Adapted from Bäck et al.[28].

In human coronary artery SMCs, $LTB_4$ induces an approximately fourfold increase in whole-cell currents measured by patch clamp[28]. Similar, but lower (around 1.5-fold increase), responses are also observed after stimulation with U75302[28], a partial agonist selective for the $BLT_1$ receptor[25,57,58,59] In contrast, $LTB_4$ does not affect membrane currents in isolated membrane patches from those cells[28]. The latter observation hence excludes any direct effects of $LTB_4$ on ion channels in vascular SMCs, which have previously been described in sensory neurons[60] and cerebellar liposomes[61].

In addition to BLT receptor expression, CysLT receptors are also expressed on vascular SMCs. In human coronary artery SMCs, CysLT2, but not CysLT1, receptor mRNA have been detected by RT-PCR[62]. In the latter study, LTC4 increased intracellular calcium with an EC50 of 10.2 nM, which was inhibited by nicardipine but not by zafirlukast, montelukast, or pranlukast[62]. However, those experiments were performed at high extracellular calcium concentrations (200 mM CaCl2), which may raise a doubt as to their physiological conditions. In contrast to those findings, rat aortic SMCs express greater amounts of CysLT1 receptor protein compared with CysLT2 receptor protein, measured by Western blot[7]. In the latter cells, the intracellular calcium increase (measured in buffer containing 1.5 mM CaCl2) induced by LTD4 (EC50: 0.54 nM) was inhibited by both CysLT1 receptor antagonist montelukast and the dual CysLT1/CysLT2 receptor antagonist BAYu9773.

### Vasoconstriction

As previously mentioned, although the BLT receptors mainly have been associated with leukocytes, recent findings indicate that structural vascular cells are also activated by $LTB_4$[28,29,36,59,63]. Initial studies had failed, however, to demonstrate an $LTB_4$-induced vasoconstriction in both pulmonary[64] and systemic vessels[45,65]. In the latter studies, potential $LTB_4$-induced effects may have been masked by the use of cumulative challenge. The $BLT_1$ receptor is rapidly desensitized by its ligand in isolated cells due to phosphorylation of its cytoplasmic tail[66,67]. Likewise, the contractile response to $LTB_4$ in the guinea pig lung parenchyma is characterized by tachyphylaxis and only noncumulative challenge unmask contractile actions of $LTB_4$[59,68]. The $LTB_4$-induced contractions of the latter preparation reveal $LTB_4$ as being rather vasoactive than a bronchoconstrictor[59]. In fact, with a noncumulative approach, $LTB_4$-induced vasoconstriction has been detected in the human pulmonary artery[59], guinea-pig pulmonary artery and aorta[36,59], as well as in rat basilar artery[69]. This $LTB_4$-induced vasoconstriction is an indirect response mediated by the release of histamine and thromboxane (TX) $A_2$[36,59]. Some of the effects of $LTB_4$ in isolated vessels have been shown to be endothelium dependent[36], whereas in others, the vasoconstrictive effects of $LTB_4$ are preserved even after endothelial denudation[59], which has strengthened the notion of SMC activation by $LTB_4$.

CysLTs were early identified not only as bronchoconstrictors, but also as vasoconstrictors. In the pulmonary circulation, cysLTs induce a preferential vasoconstriction in venous compared with arterial samples derived from the human lung[44,48,49,64,70,71]. However, as will be discussed below, endothelium-derived factors play an important modulatory role of LT-induced contractions of pulmonary arteries[48], and in the absence of a functional endothelium, human pulmonary arteries display contractions similar to those observed in pulmonary veins. In contrast to the airways, contractions of isolated human pulmonary vessels are either resistant[44,48,53] or only marginally inhibited[49] by $CysLT_1$ receptor antagonists. Whereas cysLT-induced contractions of human pulmonary veins are inhibited by BAY u9773[44], pulmonary arteries are also resistant to this treatment[48,49]. These observations hence suggested the presence of an atypical CysLT receptor on human pulmonary arterial vascular smooth muscle, not fitting into the pharmacological classification as being either a $CysLT_1$ or $CysLT_2$ receptor[4].

While the initial observations proposing a third CysLT receptor in the vasculature were based on the resistance to CysLT receptor antagonists, there are also agonist data in favor of an atypical CysLT receptor. For example, in porcine and human pulmonary arteries, $LTC_4$ and $LTD_4$ induce similar contractions, whereas $LTE_4$ is inactive[49,50]. In addition, the selective $CysLT_2$ receptor agonist BAY

u9773 is ineffective as an agonist in both the porcine[50] and human[49,72] preparations. Taken together, this rank order of potency ($LTC_4$ = $LTD_4$, whereas $LTE_4$ and BAY u9773 are inactive) further supports that the receptor mediating contractions of these preparations may be different from the previously described $CysLT_1$ and $CysLT_2$ receptors ($LTD_4 > LTC_4 > LTE_4$ and $LTD_4 = LTC_4 > LTE_4$, respectively). Similar agonist characteristics have also been described for the residual $LTD_4$-induced contraction observed in the guinea pig lung parenchymal strip in the presence of $CysLT_1$ receptor antagonists[51]. These functional observations in favor of further CysLT receptor subtypes have also been supported by ligand binding studies in human lung parenchyma, indicating specific $LTC_4$ and $LTD_4$ binding sites with differential affinity for CysLT receptor antagonists[73]. Finally, the CysLT receptor associated with contractile responses of the aorta derived from Wistar rats in which diabetes is induced by streptozotocin express similar antagonist characteristics as the atypical CysLT receptors on pulmonary arteries[52]. Since the latter findings are in contrast to another report characterizing $CysLT_1$ receptor–dependent responses in Wistar rat aortas[7], it can be speculated that diabetes may influence vascular atypical CysLT receptor expression.

In human systemic vessels, there is a striking difference in contractile responses induced by cysLTs. For example, while $LTC_4$ and $LTD_4$ are almost full agonists in human saphenous veins, only small and highly variable contractions are induced by those LTs in internal mammary arteries[45,65,74]. In contrast to findings in the pulmonary circulation[48], this unresponsiveness to LTs is preserved after endothelium denudation[45,74]. Likewise, the medial layer of mammary arteries has been suggested to present less specific LT binding compared with the media of saphenous veins[45]. Such preferential vasoconstriction of saphenous veins compared with mammary arteries may affect graft patency when used in coronary artery bypass surgery.

In human intracerebral arteries, $CysLT_2$ receptor expression has been detected immunohistochemically in the SMC layer[75]. However, human superfused cerebral artery strips are unresponsive to $LTC_4$ and $LTD_4$[76]. Furthermore, although both $CysLT_1$ and $CysLT_2$ receptor mRNA have been detected in human saphenous veins by RT-PCR[47], the constriction of this vascular segment induced by $LTC_4$ and $LTD_4$ appears to be solely mediated through the $CysLT_1$ receptor[45,47,77]. Taken together, these studies suggest that, in contrast to human pulmonary veins, $CysLT_2$ receptor expression in systemic vessels may not be coupled to vasoconstriction. This notion is further supported by the lack of LT-induced coronary vasoconstriction *in vitro*[46,78], although $CysLT_2$ receptor mRNA expression can be detected in coronary artery SMCs[62]. In contrast, atherosclerotic coronary arteries contract in response to cysLTs[46,78], suggesting increased sensitivity to LTs during atherogenesis. The reason for this change of reactivity has not been completely examined, but it has been noted that the number of LT binding sites are increased in atherosclerotic vessels[16,78]. Interestingly, the contractile response induced by $LTC_4$ in human atherosclerotic coronary arteries is inhibited by the $CysLT_1$ receptor antagonist ICI198615[46]. Furthermore, atherosclerotic lesions from human carotid arteries display threefold higher levels of $CysLT_1$ receptor transcripts compared with $CysLT_2$ receptor transcripts[56]. Taken together, those findings suggest a major role of the $CysLT_1$ receptor in atherosclerosis. It is in this intriguing context that $ApoE^{-/-}$ mice display an increased $CysLT_1$ receptor expression in the aorta compared with nonatherosclerotic mice[79], although the localization of the latter receptor up-regulation remains to be established.

The effects of intracoronary $LTD_4$ administration has been evaluated in a study of a small number (n = 6) of patients[80]. While all patients examined in that study were devoid of significant stenosis on coronary angiogram, the cardiovascular risk factors are unknown and coronary atherosclerosis cannot be completely excluded. The coronary vascular resistance was calculated based on thermodilution measurements of coronary blood flow. Unchanged during the intracoronary injection of a bolus of $LTD_4$ (3 nmol), the coronary vascular resistance increased at 10 and 15 min after administration. At the same time, systemic mean arterial blood pressure initially decreased while heart rate was increased, returning to baseline after 10 and 1 min postinjection, respectively[80]. Although these effects may have been indirectly mediated through $LTD_4$-induced increase of circulating levels of catecholamines[80], the latter study supports significant hemodynamic effects induced by the LTs.

### SMC Proliferation and Migration

Rat aortic SMC DNA synthesis and cell count are increased in the presence of $LTB_4$, $LTC_4$, and $LTD_4$, whereas $LTE_4$ is inactive[81,82,83]. The effects of $LTC_4$ and $LTD_4$ in rat aortic SMCs are inhibited by either FPL55712 or ICI198615, suggesting a $CysLT_1$ receptor–mediated SMC proliferation[84]. Results obtained with $LTB_4$ in human and rat vascular SMCs support that $LTB_4$-induced SMC proliferation is mediated through the $BLT_1$ receptor[28,85].

The notion of $LTB_4$ being a neutrophil chemoattractant has led to the hypothesis that other cells would also migrate in response to BLT receptor activation. While this notion initially had been tested on rat SMCs[85,86], the first evidence of $LTB_4$-induced chemotaxis of human SMCs were derived from migration of human coronary artery SMCs[28]. The maximum migration induced by $LTB_4$ in the latter study was approximately 50% greater compared with recombinant platelet-derived growth factor BB-homodimer (PDGF), a known SMC chemoattractant. Furthermore, $LTB_4$-induced SMC chemotaxis is inhibited by both ONO4057 and the selective $BLT_1$ receptor partial agonist U75303, hence correlating the functional responses to the detected $BLT_1$ receptor mRNA and protein in these cells[28]. Also $LTC_4$ induce migration of human coronary artery SMCs[62], but since no antagonist data have been provided, the CysLT receptor associated with this response remains to be established.

### SMC and Atherosclerosis

Proliferation and migration of vascular SMCs lead to intimal hyperplasia and fibrous cap formation, which are key features of atherosclerosis[87]. Interventions within the LT pathway in different mouse models of atherosclerosis have yielded contradictory findings, however, on atherosclerotic lesion SMC content. For example, $ApoE^{-/-}$ mice with a targeted $BLT_1$ receptor have fewer SMCs in their lesions at 10 weeks of age[29], whereas pharmacological inhibition of LT formation in $ApoE^{-/-}$x$LDLR^{-/-}$ mice leads to increased lesion SMC content at 6 months of age[88]. These differences may indicate that LT synthesis inhibition exerts differential effects on SMCs compared with BLT receptor targeting. It is not to be excluded, however, that the apparent differences between the latter two studies were due to the different mouse strains and time points studied. In favor of a time dependency of the response, it can be noted that the effects of either genetic or pharmacological targeting of the $BLT_1$ receptor in atherosclerotic mice induces predominant beneficial effects in younger animals with less complicated lesions[89,90]

### Intimal Hyperplasia and Restenosis

Proliferation and migration of SMCs are also key features of the intimal hyperplasia that causes restenosis after vascular interventions[91]. Although the use of stents has reduced the incidence of restenosis after coronary angioplasty, this complication still remains a substantial clinical problem, with intimal hyperplasia being the predominant mechanism of restenosis after stenting[91]. Interestingly, angioplasty is a stimulus for intracoronary formation of lipoxygenase products[92]. Together with the above-mentioned potent effects of the LTs on SMC proliferation and migration, these observations have provided a rationale for evaluating LT modifiers in models of restenosis.

Treatment with the BLT receptor antagonist BIIL284 during 14 days following balloon injury of the carotid artery in rats has been shown to prevent intimal hyperplasia[40]. Furthermore, the LT synthesis inhibitor MK886 reduces intimal hyperplasia in a model of photochemical injury of the rat femoral artery[85]. In the latter study, the finding that the BLT receptor antagonist ONO4057, but not the $CysLT_1$ receptor antagonist ONO1078, had beneficial effects led the authors to conclude that $LTB_4$, but not cysLTs, are involved in intimal hyperplasia. However, the reduction in intima area induced by BLT receptor antagonism was substantially weaker (approximately 20% reduction) compared with effects of MK886 (approximately 40% reduction) in the photochemical injury[85]. This is in contrast to the pronounced effects of BLT receptor antagonism in preventing intimal hyperplasia after balloon injury (approximately 50% reduction)[28]. Taken together, those studies suggest that direct stimulation of the vascular SMC layer by balloon injury may preferentially activate the BLT receptor pathway. Although the $CysLT_1$ receptor antagonist ONO1078 did not prevent intimal hyperplasia in the photochemical injury model[85], it cannot be excluded that cysLTs potentially are involved in this response through activation of the $CysLT_2$ receptor. In addition, another selective $CysLT_1$ receptor antagonist (MK571) has been shown to inhibit intimal hyperplasia after balloon injury[81]. Again, whether or not this represents a difference between endothelial and SMC injury as stimulus for the intimal hyperplasia, remains to be established.

In addition to direct effects on the smooth muscle layer, angioplasty triggers an inflammatory response with leukocyte activation, which potentially could be mediated through $LTB_4$ signaling. In fact, both the nonspecific cyclo- and lipoxygenase inhibitor BW755C[93] and the FLAP-inhibitor MK886[94] decrease neutrophil deposition at sites of arterial injury in pigs. Although the effects on intimal hyperplasia were not evaluated in the latter studies, the beneficial effects of inhibiting the LT signaling in different models of angioplasty[28,81,85,93,94] suggest that both direct effects on vascular SMCs and anti-inflammatory effects through inhibition of leukocyte recruitment may be involved.

Although no systemic treatment hitherto has proved to reduce the rate of coronary restenosis in humans, targeting LT signaling could potentially be used as a therapeutic strategy in preventing coronary reocclusion after angioplasty. BLT receptor antagonists could represent either an alternative or a complement to the present use of locally administered rapamycin derivates by means of drug eluting stents (DES). Although the use of DES in interventional cardiology has led to a decreased risk of restenosis, recent clinical data have suggested an increased risk of late stent thrombosis following DES implantation[95]. One possible explanation for an increased thrombotic risk could be that the cell cycle inhibitors block not only SMC proliferation, but also re-endothelialization and the covering of the stent. Exposure of a metal stent in the coronary vessel is a stimulus for platelet activation and aggregation. No study has evaluated the effects of LT inhibitors on re-endothelialization after angioplasty, and the effects of LT inhibitors in this context are difficult to foresee. LT receptors are indeed expressed on the endothelium and potential LT-induced effects on ECs will be addressed in the next part of the present review.

### LT RECEPTORS ON EC

#### Expression and LT signaling in ECs

In human vessels, endothelial expression of $BLT_1$ receptors is observed only in atherosclerotic and not in healthy arteries, suggesting an induction during atherogenesis (Fig. 2)[40]. The latter finding is hence consistent with the proinflammatory regulation of $BLT_1$ receptor transcription in the vascular wall discussed above. Levels of intracellular calcium have been reported to be either modestly increased (8%[96]) or unchanged[97] after $LTB_4$ challenge of HUVECs. Although incubation with either LPS or cytokines has been proposed to increase BLT receptor–mediated calcium responses in HUVECs, the lack of statistical evaluation of the data in that study makes those results difficult to interpret[98]. In contrast, *all-trans* retinoic acid induces significantly increased $BLT_1$ receptor mRNA levels, as well as significantly enhanced endothelium-dependent vascular responses in the guinea pig aorta[36], supporting enhanced functional responses as a consequence of endothelial BLT receptor induction.

The first study to examine CysLT receptor mRNA levels in HUVECs reported that mRNA for the $CysLT_2$, but not $CysLT_1$, receptor was detected by RT-PCR[99]. A subsequent report confirmed this finding and, in addition, showed that $CysLT_2$ receptor transcript levels decreased with increasing passage numbers[56], proving a possible explanation as to some of the conflicting results obtained in studies of LT-induced effects in HUVECs. Although $CysLT_2$ receptor mRNA also has been detected in human coronary artery ECs[62], those findings do not exclude a role for $CysLT_1$ receptor signaling in ECs. In fact, $CysLT_1$ receptor expression can be induced in HUVECs after 24-h stimulation by IL-1β (Fig. 3)[55].

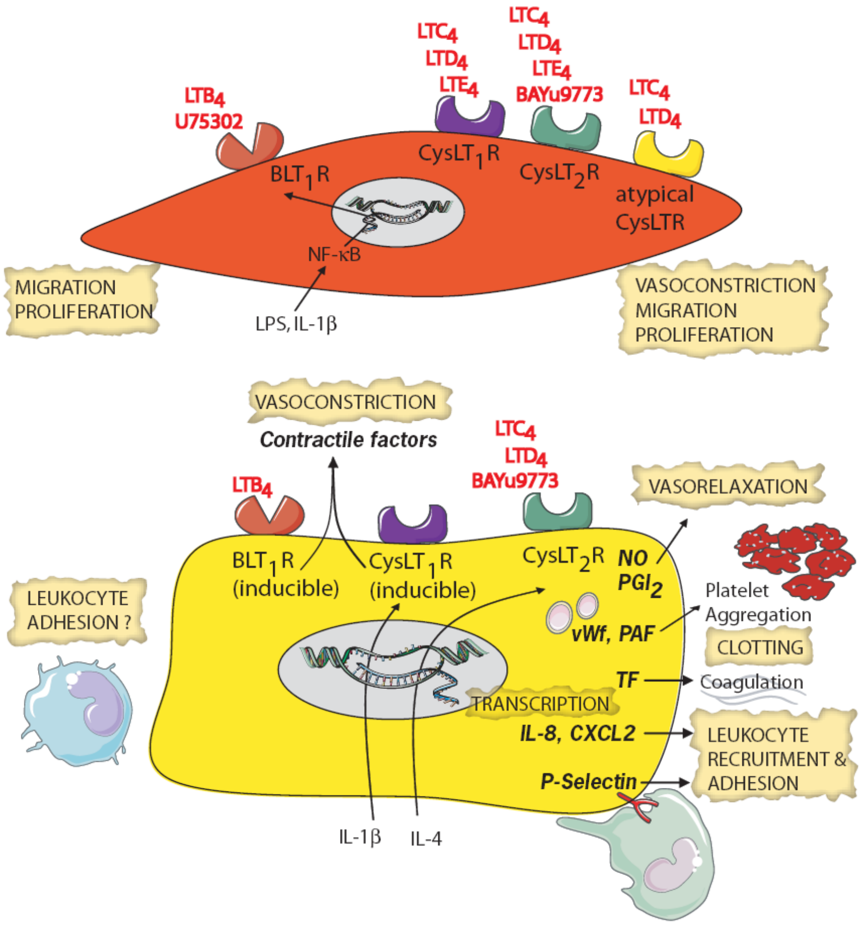

**FIGURE 3.** Schematic drawing of LT receptor expression and signaling on vascular SMCs (top) and ECs (bottom).

Furthermore, studies of human brain tissue have revealed $CysLT_1$ receptor immunostaining in microvascular ECs[100], while the $CysLT_2$ receptor was not detected in these cells[75]. However, a time-dependent increase of $CysLT_2$ receptor protein is observed in microvascular ECs after brain injury[75]. Since $CysLT_2$ receptor up-regulation was most prominent in the regenerated microvascular ECs, while no $CysLT_2$ receptor–positive cells were found in the necrotic regions, these findings suggested an inducible expression[75]. *In vitro* studies of HUVECs have indicated that the $CysLT_2$ receptor expression is highly dependent on transcriptional regulation, being up-regulated by IL-4 in a dose- and time-dependent manner, and down-regulated by TNFα[56].

Challenge of HUVECs with $LTC_4$, $LTD_4$, or the selective $CysLT_2$ receptor agonist BAY u9773 increases intracellular calcium concentrations. The pattern of this cysLT-induced response has been described as an initial rapid rise in intracellular calcium, followed by an oscillatory response[56,101]. Removal of external calcium does not affect the pattern of responses[101]. The intracellular calcium chelator BAPTA-AM eliminated $LTC_4$- and $D_4$-induced transients[101], whereas EGTA did not prevent the initial rise in calcium, but led to a cessation of ongoing oscillations[56]. Studies of bovine aortic ECs have shown $[^3H]LTC_4$ binding, which is only weakly displaced by FPL55712[102,103,104]. In cultured monolayers, the $K_d$ for $[^3H]LTC_4$ binding was 6.8 n*M*[104], of which 33% was associated with the cell surface membrane fraction and 25% associated with subcellular membrane fraction, with identical $K_d$ values[104]. These observations suggest a subcellular distribution of $LTC_4$ binding sites, with similar weak inhibition by FPL55712[104]. In colon epithelial cells, a nuclear localization of CysLT receptors has been described[105], but it is presently premature to speculate on CysLT receptor expression on the EC nuclear membranes. It cannot be excluded that the reported subcellular $[^3H]LTC_4$ binding[104] represents LT-binding structures other than receptors.

### Endothelial-Dependent Release of Vasoactive Factors by LTs

While the contraction of the guinea pig pulmonary artery induced by $LTB_4$ is unaltered by endothelial denudation[59], the $LTB_4$-induced responses in the aorta from the same species are almost completely endothelium dependent[36]. The main part of endothelial $BLT_1$ receptor activation in the latter model is mediated through release of histamine. Also, thromboxane and other metabolites of the cyclooxygenase pathway have been detected in supernatants from either endothelium-intact vascular preparations or cultured HUVECs after $LTB_4$ challenge[36,106]. However, in bovine aortic ECs, $LTD_4$ and $LTC_4$, but not $LTB_4$, increase $PLA_2$ activity and 6-keto$PGF_{1\alpha}$ release, suggesting a CysLT receptor–induced effect[107,108]. Furthermore, HUVECs release radiolabeled AA after cysLT stimulation[109,110], leading to prostacyclin formation[109,110,111,112]. Prostacyclin, measured as 6-keto$PGF_1\alpha$, constitutes 62% of the AA metabolites formed after cysLT stimulation of HUVECs, followed by $PGF_2\alpha$ (12%) and $PGE_2$ (2–4%)[109]. The prostacyclin release induced by $LTC_4$ reaches maximum at 15–30 min, which is slower than that induced by thrombin[109] and histamine (maximal release within 10 min)[109,110,112]. Several studies have described a more pronounced arachidonic release and prostacyclin formation by $LTC_4$ as compared with $LTD_4$, whereas $LTE_4$ is inactive[106,111,112]. The available antagonist data on inhibition of LT-induced AA metabolism in HUVECs are conflicting, however. For example, the nonspecific $CysLT_1$ receptor antagonist FPL55712 has been reported both to inhibit 6-keto$PGI_2\alpha$ release[112] and not to block the release of radiolabelled AA[109] after $LTC_4$ challenge.

The release of cyclooxygenase products in response to LT challenge may be involved in the regulation of pulmonary hemodynamics[113]. CysLT formation has been demonstrated in the human pulmonary circulation[114] and elevated levels of cysLTs are detected in bronchoalveolar lavage fluid from infants with pulmonary hypertension[115]. Those findings have led to the suggestion that cysLTs may be involved in the pathophysiology of pulmonary hypertension. However, based on the *in vitro* findings in the human pulmonary artery[50], the question can be raised as to what is the role of cysLTs in the pulmonary circulation. Isolated human pulmonary arteries are contracted by $LTC_4$ and $LTD_4$[48,49,53], which supports the hypothesis that cysLTs may increase pulmonary arterial pressure in humans. On the other hand, $LTC_4$ challenge leads to release of prostacyclin at concentrations sufficient to functionally antagonize the pulmonary arterial contractions[48]. The relaxant effect of prostacyclin has a beneficial role in pulmonary hypertension and is used as treatment of this disease[116]. While a definitive role for cysLTs (constriction and/or dilatation) in pulmonary vessels under physiological conditions is difficult to establish, the findings in isolated vessels[48] suggest that the degree of endothelial function may be decisive on the cysLT vasomotor responses *in vivo*. In support of the latter notion, human 5-LO overexpression by adenoviral gene transfer in rat lungs induces pulmonary hypertension only in combination with endothelial injury by monocrotalin[117]. Furthermore, the LT synthesis inhibitors zileuton and MK886 prevent monocrotalin-induced pulmonary hypertension[117].

In contrast to pulmonary arteries, in which endothelium-dependent LT responses mainly are mediated through cyclooxygenase activation, LTs may predominantly stimulate the NO pathway in pulmonary venous ECs. For example, LT-induced contractions of porcine pulmonary veins are unmasked only after inhibition of NO synthesis, while cyclooxygease inhibition does not alter this response[118]. Endothelium-dependent relaxations induced by $LTD_4$ in human pulmonary veins are inhibited by L-NOArg, but enhanced by $CysLT_1$ receptor antagonists[119]. The latter observation provides some interesting information. First, endothelial NO release in response to cysLTs seems to be $CysLT_2$ receptor dependent (Fig. 3). Second, endothelial $CysLT_1$ activation may in contrast be linked to release of contractile factors (Fig. 3). This notion is further supported by the superimposable endothelium-dependent inhibition of $LTC_4$-induced contractions in porcine pulmonary arteries by indomethacin and $CysLT_1$ receptor antagonism[50]. Taken together, those studies provide a first suggestion of the $CysLT_1$ receptor as potential inducer of endothelial dysfunction. In contrast, it can be speculated that the $CysLT_2$ receptor–induced NO release could be beneficial in this context, which is supported by the coincidence of increased NO production and diminished systemic pressor response to $LTC_4$ after directed EC expression of the human $CysLT_2$ receptor in mice[97]. Finally, NO may also act as feedback mechanism leading to decreased LT formation, as recently suggested by studies of allergen-induced reactions[120]

### Endothelial LT Receptors in Platelet Aggregation and Coagulation

$LTC_4$ and $LTD_4$, but not $LTB_4$, stimulate von Willebrand factor exocytosis from endothelial Weibel-Palade bodies (Fig. 3)[101]. Together with the increased secretion of both thromboxane[59] and platelet activating factor[121], those observations suggest a prothrombotic LT-induced response, indirectly mediated through structural components of the vascular wall. These LT-induced effects on ECs may provide a potential link between inflammation and atherothrombosis. The LT receptor(s) associated with this prothrombotic response, however, remains to be established. For example, although the $CysLT_1$ receptor antagonist pobilukast decreases the $LTD_4$-induced vWF secretion, this inhibition occurs only at antagonist concentrations substantially higher (3 and 10 $\mu M$) compared with those needed to inhibit LT-induced bronchoconstriction[101]. In addition to platelet aggregation, $LTD_4$ may also be procoagulant. Stimulation of HUVECs with $LTD_4$ increases tissue factor (TF) transcript- and protein levels, associated with activation of factor Xa through the extrinsic coagulation pathway (Fig. 3)[122].

### LT Receptors in EC-Leukocyte Interactions

While leukocyte recruitment by $LTB_4$ mainly is mediated through direct activation of BLT receptors expressed on, for example, monocytes, neutrophils, and T cells[18], $LTB_4$-induced endothelial activation has also been implicated in increased leukocyte adherence[123]. However, the *in vitro* neutrophil adherence observed after $LTB_4$ stimulation of HUVECs is weak[96,124] and, in addition, similar to basal values of adherence[96]. The role of endothelial BLT receptors in leukocyte adhesion is hence not fully understood and negative findings have also been reported. For example, $LTB_4$ has no effect on P-selectin expression in HUVECs[125] and a recent study showed no effect on monocyte arrest under physiological flow conditions after stimulation of ECs with $LTB_4$[126]. In contrast, cysLT-induced leukocyte adherence may be predominantly dependent on stimulation of ECs rather than direct effects on leukocytes. While cysLTs alone do not stimulate neutrophil aggregation, ECs stimulated with, and then washed free of, $LTC_4$ exhibit hyperadherence to neutrophils[121]. Furthermore, $LTC_4$ and $LTD_4$ increase endothelial binding of anti-P-selectin antibodies, whereas $LTE_4$ is significantly less active (Fig. 3)[125,127]. The endothelial P-selectin increase is not inhibited by the $CysLT_1$ receptor antagonists pranlukast, zafirlukast, or pobilukast, supporting a $CysLT_2$ receptor–induced effect (Fig. 3). While maximal P-selectin surface expression occurs within 10 min after stimulation with either $LTC_4$ or $LTD_4$[127], $CysLT_2$ receptor activation may also induce prolonged proinflammatory effects through increased transcriptional activity in ECs[122]. The genes up-regulated by $LTD_4$ are largely the same as those induced by thrombin stimulation of HUVECs, suggesting potential synergistic effects between $CysLT_2$ and protease-activated (PAR-1) receptors on ECs[122]. The CXC chemokines CXCL-2[79] and IL-8[122] are found among the most up-regulated genes after 1-h stimulation of HUVECs with $LTD_4$. Taken together, these data support a role for endothelial $CysLT_2$ receptors in leukocyte recruitment and adhesion (Fig. 3). However, CysLT receptors are expressed also on leukocytes[18], and the exact interaction between inflammatory cells and ECs in LT-induced leukocyte recruitment remains to be established.

### CONCLUSION

The local production of LTs in the vicinity of the vascular wall in combination with the expression of LT receptors on vascular SMCs and ECs suggest a crucial role of para- and autocrine LT signaling in vascular inflammatory reactions. As described in the present review, some of the LT-induced responses in the vascular wall are shared between BLT and CysLT receptors, whereas others exhibit differential and even opposing effects, depending on which receptor subtype is activated. Furthermore, the same receptor subtype may induce differential effects depending in what cell the receptor is activated. Finally, transcriptional regulation of LT receptor expression may change the receptor subtypes expressed at different time points and under different physiological and pathophysiological conditions. In conclusion, mechanistic studies elucidating the pharmacology and physiology of LT receptor signaling within the vascular wall is of utter importance to identify potential therapeutic targets within the LT pathway for the treatment of cardiovascular disease.

### ACKNOWLEDGMENTS

The author is supported by the Swedish Heart and Lung Foundation, the CMM Foundation, Torsten and Ragnar Söderberg Foundation, and Karolinska Institutet.
